# Foodborne Helminths in Imported Fish: Molecular Evidence from Fish Products in the Kazakhstan Market

**DOI:** 10.3390/foods14203466

**Published:** 2025-10-11

**Authors:** Ainura Smagulova, Aitbay Bulashev, Karina Jazina, Rabiga Uakhit, Lyudmila Lider, Aiganym Bekenova, Dana Valeeva, Vladimir Kiyan

**Affiliations:** 1 Laboratory of Biodiversity and Genetic Resources, National Center for Biotechnology, Astana 010000, Kazakhstan; smagulova0114@gmail.com (A.S.); dzhazinak01@mail.ru (K.J.); erken.uakhitrabiga@gmail.com (R.U.); 2 Department of Veterinary Medicine, S. Seifullin Kazakh Agrotechnical Research University, Astana 010000, Kazakhstan; aytbay57@mail.ru (A.B.); l.lider@kazatu.kz (L.L.); aiganym5555@mail.ru (A.B.); valeyeva.dana2002@mail.ru (D.V.); 3 Scientific Center for Biological Research, Astana 010000, Kazakhstan

**Keywords:** imported seafood, fish-borne parasites, *Anisakis* spp., molecular diagnostics, helminths, DNA sequencing, food safety, Kazakhstan

## Abstract

The increasing reliance on imported fish products in Kazakhstan raises concerns about the presence of fish-borne parasitic infections, particularly zoonotic helminths that pose risks to public health. This study aimed to assess the diversity and prevalence of helminths in commercially imported marine fish using both traditional and molecular diagnostic methods. A total of 670 specimens representing 17 fish species were collected from retail markets in Astana, Almaty, and Karaganda. Macroscopic inspection and muscle compression techniques were used to detect larval parasites, followed by DNA extraction and PCR amplification targeting the *ITS*-2, 5.8S, 18S rRNA, and mitochondrial *COX* gene regions. Sequencing and phylogenetic analysis confirmed the presence of cestodes (*Eubothrium crassum*, *Hepatoxylon trichiuri*, *Nybelinia surmenicola*), acanthocephalans (*Echinorhynchus gadi*), and nematodes, with a predominance of zoonotic species from the Anisakidae family, including *Anisakis simplex*, *A. pegreffii*, *Pseudoterranova decipiens*, and *Contracaecum osculatum*. The highest levels of infection were detected in Atka mackerel (97.1%), herring (96.0%), mackerel (92.0%), and blue whiting (88.1%), while the lowest rates were recorded in smelt (6.8%), flounder (10.2%), and haddock (16.0%). This is the first molecular-based survey of fish helminths in Kazakhstan and highlights the need to integrate genetic screening into food safety control systems to better protect consumers and improve parasite monitoring of imported seafood.

## 1. Introduction

Fish are one of the primary sources of food worldwide, accounting for approximately 185.4 million tons annually [1]. The major fishing areas include the Atlantic Ocean and the Mediterranean Sea (20.9 million tons), the Indian Ocean (11.8 million tons), and the Pacific Ocean (46.4 million tons) [2]. Fish frequently serve as hosts for a wide range of parasites from various taxonomic groups, including nematodes, cestodes, trematodes, and protozoans. Parasitic infections are more common in wild fish than in aquaculture and can significantly affect fish growth, development, and overall health [3]. In Norwegian waters, nematodes from the family Anisakidae, such as *Anisakis simplex*, *Pseudoterranova decipiens*, and *Contracaecum osculatum*, are commonly found in commercially important species such as cod, haddock, char, and tuna [4,5,6]. In one study alone, 19 helminth species were identified in southeastern Norway, including 1 monogenean, 8 digeneans, 1 cestode, 2 nematodes, and 2 parasitic crustaceans [7]. Additional studies have also confirmed the presence of various other parasitic groups in this region, including cestodes, trematodes, acanthocephalans, and protozoans [8,9,10,11].

A similar situation is observed in Russian waters of the Far East and along the Murmansk coast, where zoonotic nematodes from the Anisakidae family are widespread and frequently detected in commercially important fish species such as cod, herring, flounder, and pollock [12,13,14]. Several studies also report the presence of a wide range of other parasite taxa in marine organisms from Russian waters [15,16,17]. For example, in the Barents Sea, trematodes such as *Podocotyle atomon* and *Podocotyle reflexa*, along with acanthocephalans like *Polymorphus phippsi* and *Echinorhynchus gadi*, have been identified in gammarids, which serve as intermediate hosts for various helminths [18]. In the waters around Yuzhno-Sakhalinsk, 20 different parasite species have been found in smelt, including *Diphyllobothrium* spp., *Lecithaster gibbosus*, and *Corynosoma strumosum* [19].

In Southeast Asia, a comprehensive parasitological survey in Vietnam reported 498 parasite species associated with 225 species of fish, with *Digenea* (43%) and *Monogenea* (23.5%) being the most prevalent groups [20].

In the Mediterranean Sea, recent studies have revealed a high biodiversity of fish parasites, with economically significant species such as the polyopisthocotylean *Sparicotyle chrysophrii* and the myxozoan *Enteromyxum leei* dominating infection profiles in aquaculture settings [21].

According to recent studies, a total of 78 parasite species, 44 endoparasites and 34 ectoparasites, have been identified in 10 species of horse mackerel in the South Pacific Ocean, with monogeneans being the predominant group [22]. In addition, a wide variety of other taxonomic groups has been recorded, including cestodes, nematodes, trematodes, and ectoparasitic crustaceans, highlighting the high level of biological diversity in the parasite fauna of this region [23,24,25,26]. This richness reflects the complexity of host–parasite systems and underscores the potential ecological role of parasites within marine food webs.

Parasitic infestations have a significant negative impact on the overall condition of fish by disrupting physiological processes, damaging tissues, and reducing resistance to environmental stressors [27,28,29,30]. One of the most dangerous parasites is *Anisakis* spp., a nematode whose larvae localize in the digestive tract, liver, musculature, and other internal organs. The presence of these parasites induces severe inflammatory responses, including granuloma formation and necrotic tissue changes [31]. Infected fish often exhibit emaciation, reduced motor activity, and impaired feeding behavior. Moreover, tissue damage, particularly to muscles and internal organs, can impair buoyancy and coordination, making fish more susceptible to predation [32,33,34]. Another harmful parasite, the cestode *Eubothrium crassum*, also negatively affects fish health by causing inflammatory changes in the intestinal tract. These changes are accompanied by epithelial damage, disruption of endocrine regulation, and a general decline in physical condition [35,36]. Such pathological effects not only compromise the health of individual fish but can also significantly affect population-level health, particularly under intensive aquaculture conditions.

Some of these parasitic species are zoonotic, posing significant risks to human health through the consumption of raw or undercooked fish. In this context, it is crucial to consider the parasitological status of imported fish products in Kazakhstan. Numerous parasitological studies carried out in Norway and Russia, two of the primary exporters of marine fish to Kazakhstan, have reported a broad spectrum of helminth species in commercially important fish [4,5,15,16,17,37,38]. These findings underline the importance of parasite monitoring and molecular identification as tools to ensure food safety and prevent fish-borne zoonoses in the region.

Despite the known presence of parasitic helminths in fish from exporting countries, molecular identification techniques, capable of accurately determining parasites at the species level, are rarely employed during sanitary inspections in Kazakhstan. This methodological gap poses a potential threat to consumer health and local biosecurity, especially considering that some parasitic species may be introduced into new ecosystems through imported fish or fish processing waste [39]. To date, no large-scale studies have been conducted to assess the species composition or prevalence of helminths in imported fish in Kazakhstan, leaving a significant gap in national food safety risk assessment and regulatory frameworks. This study represents the first comprehensive parasitological and molecular survey of imported marine fish sold on the Kazakhstan market. The application of molecular genetics methods enhances diagnostic precision and provides essential data on the presence of zoonotic helminths in fish products, thereby supporting more effective monitoring, risk evaluation, and food safety management.

## 2. Materials and Methods

### 2.1. Sample and Data Collection

Frozen fish samples were collected from retail fish markets in three major regions of Kazakhstan (Astana, Almaty, and Karaganda) where imported seafood is regularly supplied and sold. Morphometric parameters and origin of the fish species presented in Table 1.

The following species were analyzed (number of specimens in parentheses): herring (*Clupea harengus*, n = 50), pollock (*Theragra chalcogramma*, n = 50), mackerel (*Scomber scombrus*, n = 50), capelin (*Mallotus villosus*, n = 71), sea bass (*Dicentrarchus labrax*, n = 35), hake (*Merluccius merluccius*, n = 50), flounder (*Platichthys flesus*, n = 50), cod (*Gadus morhua*, n = 10), blue whiting (*Micromesistius poutassou*, n = 59), greenling (*Hexagrammos stelleri*, n = 45), smelt (*Osmerus eperlanus*, n = 44), char (*Salvelinus alpinus*, n = 50), haddock (*Melanogrammus aeglefinus*, n = 20), dorado (*Sparus aurata*, n = 22), amberjack (*Seriola dumerili*, n = 32), sprat (*Sprattus sprattus*, n = 76), and pink salmon (*Oncorhynchus gorbuscha*, n = 4). The collection includes representatives of three major parasitic groups: nematodes (Nematoda), tapeworms (Cestoda), and thorny-headed worms (Acanthocephala).

### 2.2. Macroscopic Examination

A preliminary visual inspection of the external surfaces of each fish was performed without magnification to assess general condition and to detect potential signs of parasitic infestation [40]. The fish were then dissected by making an incision starting from the anal opening and extending cranially along the ventral surface toward the gill chamber. Additionally, two lateral incisions were made to expose the body cavity. The alimentary canal, liver, swim bladder, and other internal organs were examined for the presence of visible adult parasites and encysted metacercariae [41]. All extracted helminths were carefully washed in distilled water and preserved in 70% ethanol for further morphological and genetic analysis.

### 2.3. Examination of Fish Muscle Using the Compression Technique

To detect encysted metacercariae in muscle tissue, small muscle samples were taken from the head, trunk, and tail regions of each fish. Each sample was placed between two glass slides and gently compressed to form a thin layer. The compressed samples were examined under a light microscope at appropriate magnifications to identify any larval helminths [42].

### 2.4. DNA Extraction

Genomic DNA was extracted from individual parasite specimens using the phenol–chloroform method. Pre-homogenized samples were incubated for 2 h at 56 °C in lysis buffer (50 mM Tris-HCl pH 8.0, 100 mM NaCl, 10 mM EDTA, 0.5% NP-40) with the addition of 5 μL of Proteinase K (20 ng/μL). DNA was precipitated with ethanol, purified, dissolved in 1× TE buffer, and stored at −20 °C until further analysis [43]. DNA concentration and purity were assessed using a Nanodrop™ One spectrophotometer (Thermo Fisher Scientific, Waltham, MA, USA).

### 2.5. PCR and Sequencing

Polymerase chain reaction (PCR) was performed using conserved primers targeting mitochondrial and ribosomal gene regions. Six pairs of primers were employed: for nematodes—NC13/NC2, NC5/NC2, SSU_F_04/SSU_22_R; for cestodes—JB3/JB4.5 and Nyb-28S-F/Nyb-28S-R; and for acanthocephalans—AcanCoI_F/AcanCoI_R. PCR amplification was carried out using HS-Taq PCR Biomasters (2×) (Biolabmix LLC, Novosibirsk, Russia), with 10 pmol of each primer and 100 ng/μL of larval DNA as template. Primer design and PCR parameters are detailed in Table 2. Amplification products were visualized by agarose gel electrophoresis and documented using a GelDoc XR+ transilluminator (Bio-Rad, Hercules, CA, USA). Positive PCR products were purified with Exonuclease I (Thermo Fisher Scientific, USA) according to the manufacturer’s instructions. Sequencing was performed on a SeqStudio Genetic Analyzer (Thermo Fisher Scientific Applied Biosystems, USA). Resulting nucleotide sequences were manually inspected and edited using BioEdit software (version 7.0) before being analyzed via BLAST against reference sequences in the GenBank database (https://www.ncbi.nlm.nih.gov/).

### 2.6. Bioinformatic Analysis

Nucleotide sequences were aligned using the MUSCLE algorithm for multiple sequence alignment, applied to partial sequences of the cox1 and nad1 mitochondrial genes. Phylogenetic trees were constructed based on a concatenated dataset using MEGA11 software (version 11) [50]. The Maximum Likelihood (ML) method was employed for tree inference, with the nearest neighbor interchange (NNI) algorithm used for heuristic search.

### 2.7. Statistical Analysis

The prevalence, mean intensity, and abundance of each ascarid species were calculated according to the methods described by Bush et al. [51].

## 3. Results

### 3.1. Import of Fish Products to Kazakhstan

**Figure 1:**
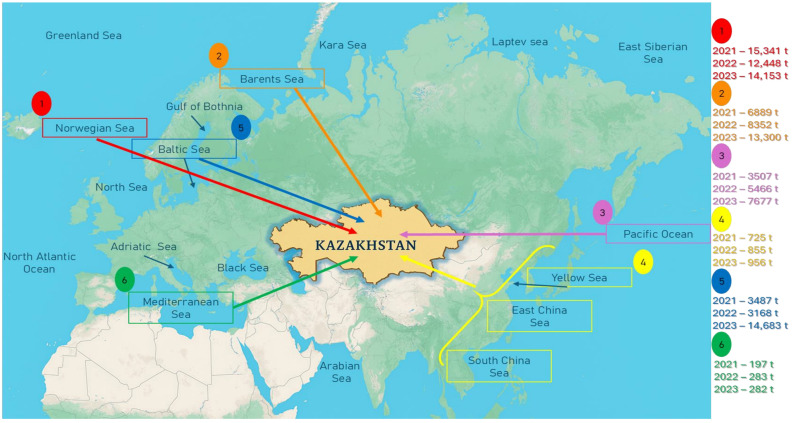
Map showing the major oceans and seas supplying imported fish to Kazakhstan. Color-coded arrows indicate trade routes along with annual import volumes (in tons) from 2021 to 2023.

Statistical data on fish imports were obtained from the Ministry of Agriculture of the Republic of Kazakhstan for the period 2021–2023. Fish and fish product imports to Kazakhstan are primarily concentrated among a limited number of supplier countries. Norway leads as the main exporter, accounting for nearly half of the total import volume. Other significant contributors include Iceland, Chile, Estonia, and countries within the Eurasian Economic Union (EAEU). Norway supplied the largest quantity, totaling 41,942 tons during this period, markedly surpassing other countries (Figure 1).

Figure 1 provides a geographic visualization of the principal marine regions from which Kazakhstan sources its imported fish. Six key regions are identified: the Norwegian Sea, Barents Sea, Pacific Ocean, East and South China Seas, Baltic Sea, and Mediterranean Sea. This data show that the Norwegian Sea consistently supplies the highest volumes of fish imports, whereas regions such as the Yellow Sea, East China Sea, and South China Sea contribute comparatively lower volumes.

### 3.2. Fish Infestation: Parasitological Analysis

Muscle tissue, visual organs, and gills were subjected to compression analysis; however, no metacercariae were detected in the examined fish samples. All identified helminthes exhibited clearly distinguishable morphological features and were identifiable through visual examination.

Parasitological examination of 19 imported fish species revealed substantial variability in both the prevalence and intensity of helminth infections (Table 3). Primary identification of parasites was conducted based on morphological characteristics specific to major taxonomic groups. Three classes of helminths were identified among the parasites recovered: Nematoda, Cestoda, and Acanthocephala.

**Figure 2:**
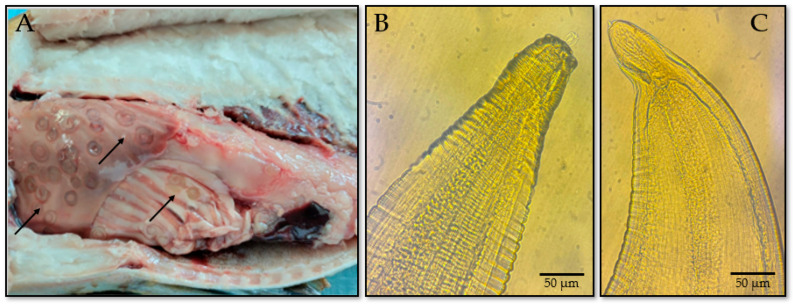
Morphological features of *Anisakis* spp. larvae identified in blue whiting (*M. poutassou*): (**A**) Larvae encapsulated on the surface of internal organs; (**B**) anterior morphology showing lips, nerve ring and cylindrical esophagus; (**C**) posterior end with cuticular lobe and terminal spine.

Nematodes were the most frequently encountered group, with morphological features enabling the preliminary identification of multiple species within the genus *Anisakis* (*Anisakis* spp., *Pseudoterranova* spp., and *Contracaecum* spp.) (Figure 2). *Anisakis* larvae were commonly found attached to the surfaces of internal organs and encapsulated within host tissue (Figure 2A). Morphologically, the larvae appeared milky white, spirally coiled, and measured 15–30 mm in length and 0.2–0.6 mm in diameter. They exhibited a blunt anterior end with three distinct lips, a cylindrical esophagus, and a dark, granular intestine (Figure 2B). A characteristic cuticular lobe was present at the posterior end, sometimes bearing a terminal spine (Figure 2C) [52,53].

**Figure 3:**
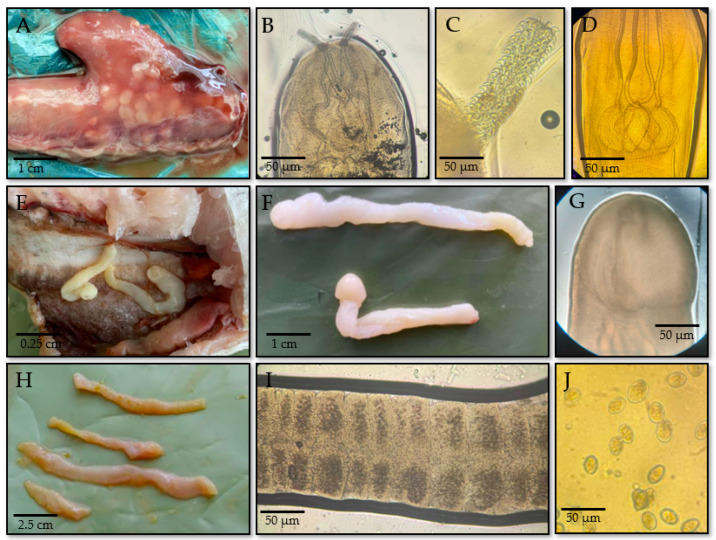
Morphological features of cestodes identified in imported fish: (**A**) *Nybelinia surmenicola* in the stomach of greenling (*H. stelleri*); (**B**) anterior end, a cuboid scolex, bearing suckers and a retractable proboscis; (**C**) proboscis with crown of hooks; (**D**) bulbous part with four muscular bulbs; (**E**) *Hepatoxylon trichiuri* in the abdominal cavity of hake (*M. merluccius*); (**F**) larval body of *H. trichiuri*; (**G**) anterior end with suckers; (**H**) *Eubothrium crassum* in the intestine of char (*S. alpinus*); (**I**) mature proglottids of *E. crassum* with developed reproductive organs; (**J**) oval eggs of *E. crassum*.

Fish were also found to be infected with various species of cestodes, primarily from the genera *Nybelinia*, *Hepatoxylon*, and *Eubothrium*, which were localized in the stomach, intestine, and abdominal cavity (Figure 3A,E,H).

The cestode *N. surmenicola* was detected in the stomach and internal organs of greenling (*H. stelleri*) and cod (*G. morhua*) (Figure 3A). The body of *N. surmenicola* is elongated, whitish, and weakly segmented, with a characteristic bladder-like posterior region. The plerocercoid measures 7–12 mm in length and 0.2–0.3 mm in width. At the anterior end, a cuboid scolex is present, bearing four suckers and a retractable proboscis (Figure 3B), which is armed with a crown of small hooks (Figure 3C). When extended, the proboscis measures approximately 0.03–0.05 mm in length. The bulbous portion of the scolex is characterized by the presence of four bulbs (Figure 3D) [54,55].

The cestode *H. trichiuri* was identified in the abdominal cavity of hake (*M. merluccius*) with a prevalence of 58% (Figure 3E). This parasite was observed in its larval stage, exhibiting an elongated, slightly curved, cylindrical, whitish body measuring approximately 8–20 mm in length and 1–3 mm in width, tapering at both ends (Figure 3F). At the anterior end, a cuboid scolex measuring 0.5–1 mm is present, equipped with four suckers (Figure 3G) and two pairs of retractable proboscises, each 0.3–0.5 mm long. These proboscises are covered with longitudinal rows of hooks, which facilitate attachment to host tissues. The posterior portion of the body is slightly swollen and contains rudimentary reproductive structures. Body segmentation is either absent or poorly developed [56].

The cestode *E. crassum* was found in the intestine of char (*S. alpinus*) with a prevalence of 42% (Figure 3H). The parasite has a ribbon-like, elongated, wide, and flattened whitish body, divided into hundreds of segments (proglottids) that gradually increase in size toward the posterior end. Adult worms typically measure 50–150 cm in length, but may reach 2–3 m, with a width of 2–5 mm. At the anterior end is an oval-shaped scolex measuring approximately 0.5–1 mm in width and 1–1.5 mm in length, equipped with two broad bothria (longitudinal suction grooves) that allow the parasite to attach to the intestinal mucosa. A narrow neck follows the scolex, leading to a series of proglottids. The proglottids are more or less square in shape, mature, and hermaphroditic; the mature segments contain a well-developed uterus with eggs and testes (Figure 3I). Nutrient absorption occurs across the entire surface of the body. The eggs are small, oval-shaped, and are released into the intestinal lumen of the host (Figure 3J). This species is commonly found in salmonids such as trout and salmon and is recognized as the causative agent of bothriasis in commercially important fish [57,58,59].

**Figure 4:**
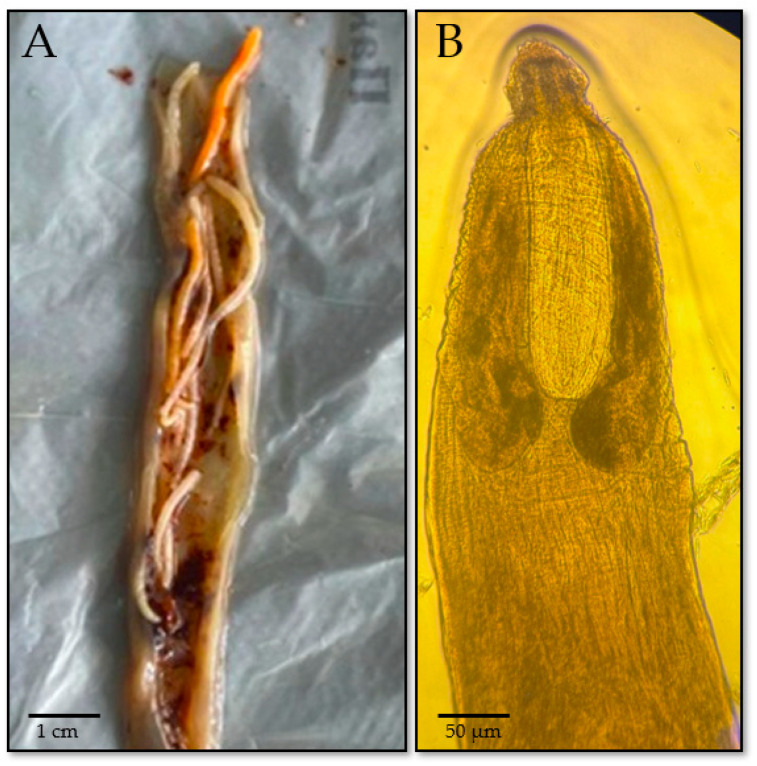
Morphological features of the acanthocephalan *Echinorhynchus gadi* identified in imported pollock (*T. chalcogramma*): (**A**) Adult worm in the intestinal tract of the host; (**B**) anterior end with spherical and visible reproductive organs in the body cavity.

The acanthocephalan *E. gadi* was identified in the gut of pollock (*T. chalcogramma*) with a prevalence of 34% (Figure 4A). The parasite has a cylindrical, slightly flattened, non-segmented body, light yellow to whitish in color, and covered with a cuticle bearing fine transverse striations. Adult specimens typically measure 20–50 mm in length, occasionally reaching up to 60 mm, with a width of approximately 1.5–3 mm. At the anterior end is a spherical, retractable proboscis (trunk) measuring about 0.5–1.0 mm, armed with several longitudinal rows of backward-curved hooks. The number and size of these hooks are species-specific and aid in parasite identification. Immediately posterior to the proboscis is the neck region, followed by the main body, which contains visible reproductive organs—the uterus in females and testes in males (Figure 4B). *Acanthocephalans* lack a digestive system; nutrient uptake occurs via osmosis through the body surface. The proboscis is used for attachment to the intestinal wall of the host, often causing mechanical injury and localized inflammation [60].

The highest prevalence was observed in greenling (*H. stelleri*) at 97.1% (95% CI: 85.1–99.9), followed closely by herring (*C. harengus*) at 96.0% (95% CI: 86.3–99.5) and mackerel (*S. scombrus*) at 92.0% (95% CI: 80.7–97.8). Similarly, blue whiting (*M. poutassou*) showed a high infection rate of 88.1% (95% CI: 77.1–95.1). In contrast, certain species demonstrated notably lower levels of helminth infestation. Smelt (*O. eperlanus*), flounder (*P. flesus*), and haddock (*M. aeglefinus*) exhibited low prevalence rates ranging from 6.8% to 16.0%. No parasitic infection were observed in sprat (*C. cultriventris*), sole (*S. solea*), sea bass (*D. labrax*), dorado (*S. aurata*) and salmon (*S. salar*). It is worth noting that the last three species were farmed in aquaculture.

### 3.3. Molecular Phylogenetic Analysis of Fish Parasites

**Figure 5:**
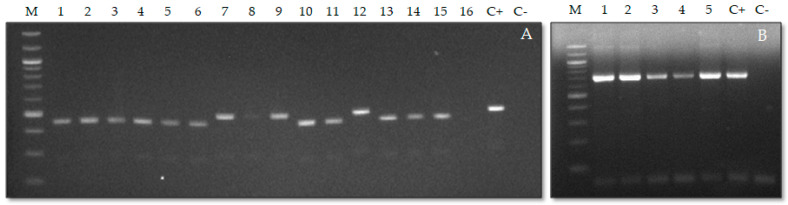
Electropherogram of gDNA PCR products of representative samples (**A**) anisakides using a universal pair of NC13/NC2 primers: lanes 1–6,10–11,13–16—DNA of *Contracaecum osculatum* samples; lanes 7–9—DNA of *Anisakis simplex* samples; lanes 12—DNA of *Hysterothylacium aduncum* samples; (**B**) acanthocephala samples using a universal pair of AcanCoI_F/AcanCoI_R primers: lane lanes 1–5—DNA of *Echinorhynchus gadi* samples. Lane M—DNA marker, lanes C+—positive control, lanes C−—negative control.

At the first stage, we performed PCR using specific primers to generate targeted amplicons, followed by sequencing. Figure 5 presents the results of PCR analysis of various parasite species using primers designed to amplify regions of the ribosomal (5.8S, *ITS2*) and mitochondrial (*COX1*) genomes.

**Figure 6:**
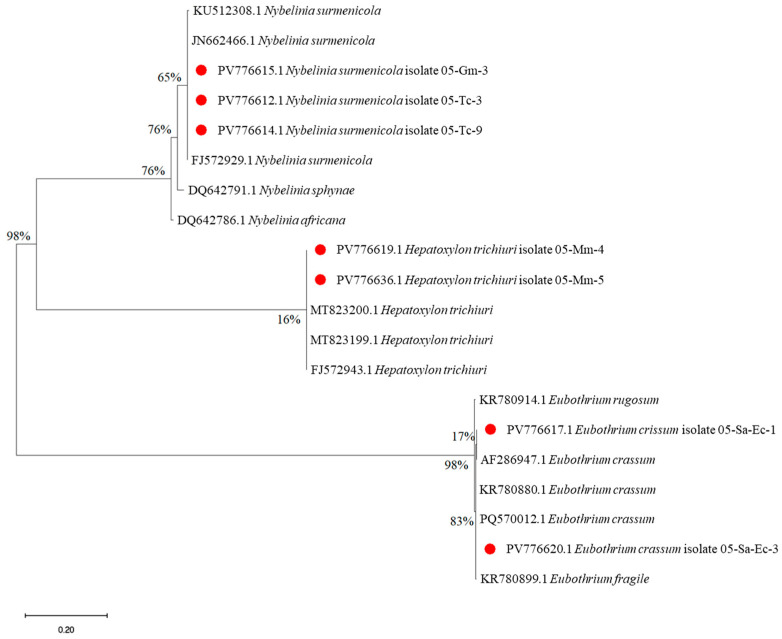
Maximum likelihood (ML) phylogram of the relationships of tapeworms (Cestoda) dataset based on LSU rDNA. Red circle—isolates found in this study.

In this study, a phylogenetic analysis of cestodes from the genera *Nybelinia*, *Hepatoxylon*, and *Eubothrium*, isolated from imported fish, was conducted for the first time using the LSU rDNA (28S) marker (Figure 6).

**Figure 7:**
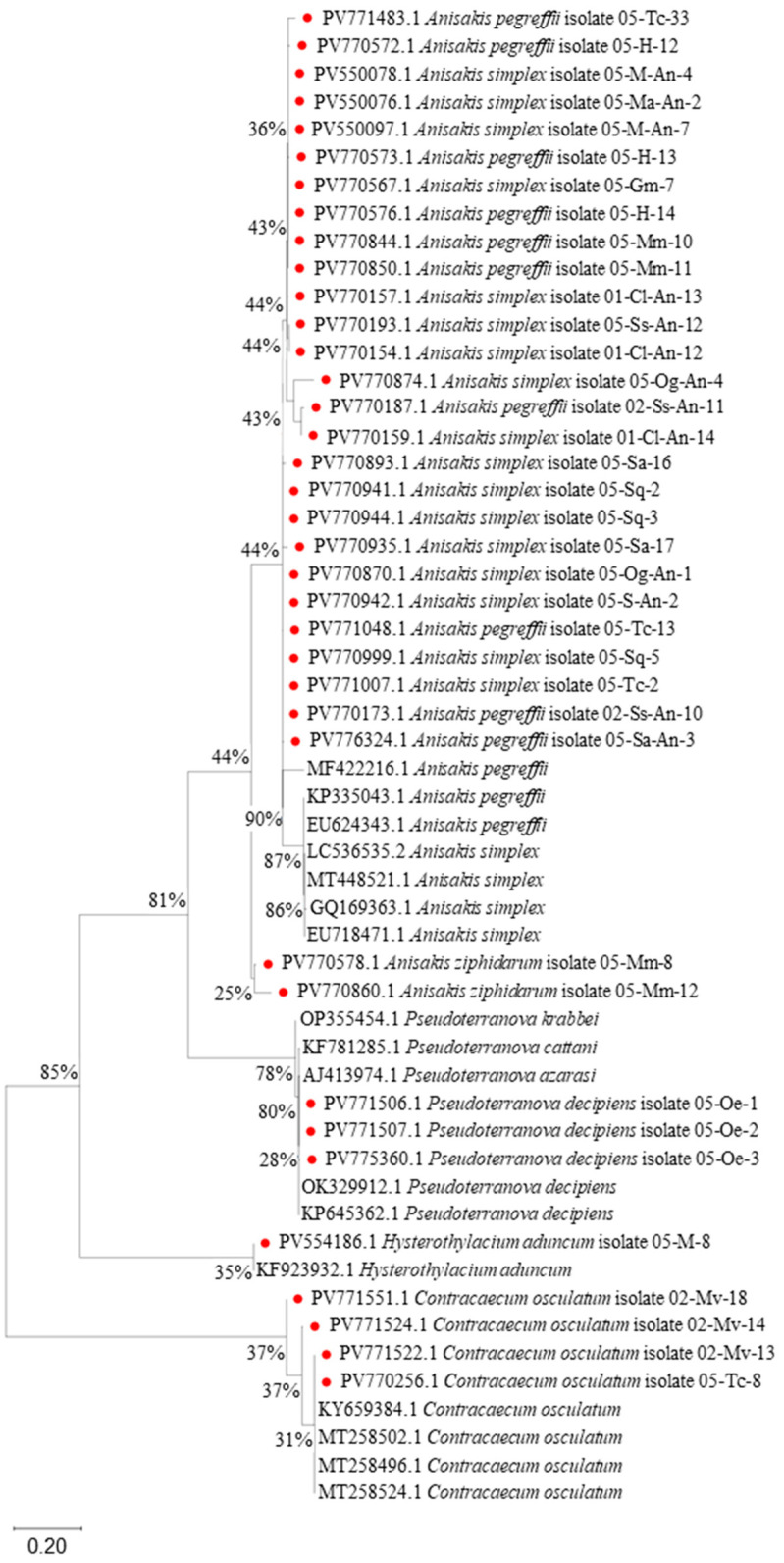
Maximum likelihood (ML) phylogram of the relationships of roundworms (Nematode) dataset based on *ITS* gene. Red circle—isolates found in this study.

Additionally, the analysis incorporated 54 nucleotide sequences of nematodes (Figure 7), with a final alignment comprising 1059 positions. The phylogenetic reconstruction confirmed the taxonomic identity of most isolates, including the detection of *A. simplex*, *A. pegreffii*, *P. decipiens*, *C. osculatum*, and *H. aduncum*. Although the *ITS* region provides only moderate resolution in differentiating closely related species (e.g., A. *simplex vs. A. pegreffii*), it remains a valuable marker for initial species identification and broader phylogenetic assessments.

**Figure 8:**
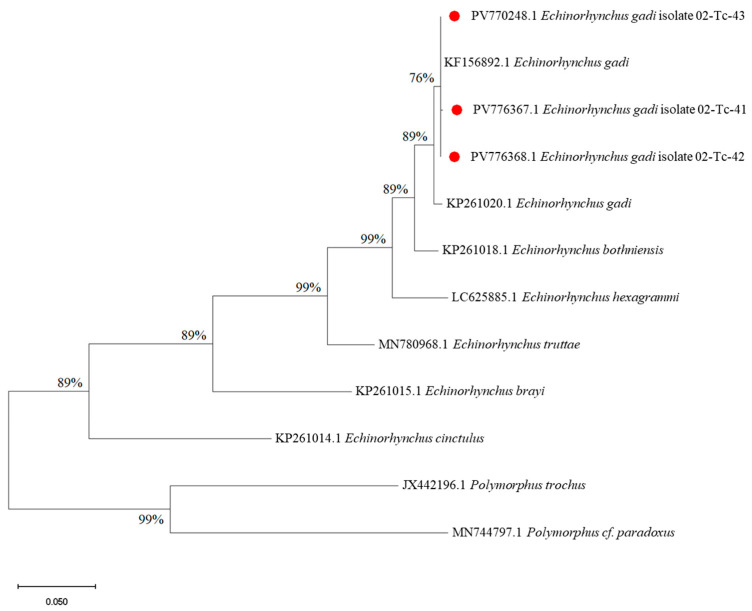
Maximum likelihood (ML) phylogenetic tree of *Echinorhynchus gadi* species. Red circle—isolates found in this study.

Furthermore, a maximum likelihood (ML) phylogenetic tree was constructed based on partial sequences of the mitochondrial *cox1* gene to determine the genetic relationships among *Echinorhynchus* spp. isolates recovered in this study (Figure 8). The analysis included 12 nucleotide sequences, aligned across 661 positions. Three isolates from this study (PV770248.1, PV776367.1, PV776368.1) clustered together with high bootstrap support (76–89%) alongside reference sequences of *Echinorhynchus gadi* (KF156892.1, KP261020.1), confirming their species identity. This clade was distinctly separated from other *Echinorhynchus* species, such as *E. bothniensis*, *E. hexagrammi*, *E. truttae*, *E. brayi*, and *E. cinctulus*. Outgroup taxa (*Polymorphus trochus* and *Polymorphus* cf. *paradoxus*) formed a clearly distinct, well-supported lineage (bootstrap 99%), affirming their separation from the *Echinorhynchus* genus.

## 4. Discussion

In Kazakhstan, as in many other countries, imported fish are routinely tested for pathogens, including parasitic helminths such as nematodes and cestodes, which pose potential risks to human health. However, diagnostic methods currently in use are often outdated, relying primarily on visual and microscopic examination. These approaches may be insufficient for detecting small, early-stage, or morphologically indistinct parasites, which can result in underdiagnosis and contribute to public health risks.

The introduction of fish-borne parasites into regions where they are poorly studied or unrecognized may lead to significant health and economic consequences. This includes potential outbreaks of zoonotic diseases and losses within the commercial fishing and food processing sectors. Given the growing dependence on imported seafood, continuous parasitological monitoring using modern molecular techniques is critical for early detection and control.

Kazakhstan primarily imports fish from Russia, Turkey, the Netherlands, France, Norway, and Iceland. According to the Bureau of National Statistics (BNS), 90% of imported fish are marine or oceanic species, such as herring, sprat, mackerel, pollock, and sea bass [61,62]. Alongside the expansion of raw material supply for local fish processing industries, the overall import volume has also increased—from 43,000 tons in 2022 to 52,600 tons in 2023, representing an 18% rise. Import substitution for these species is virtually impossible, as they do not inhabit Kazakhstan’s freshwater bodies. Moreover, artificial cultivation is either economically impractical or technically unfeasible under local conditions.

Kazakhstan’s heavy reliance on fish imports from a small number of countries increases its vulnerability to global market fluctuations and changes in international export policies. In 2021 and 2023, the dominant species on the Kazakhstani fish market were mackerel (24,806 tons), salmon (11,194 tons), and herring (10,763 tons), while other species were imported in much smaller quantities.

Freezing is a recognized method for disinfecting fish from parasite larvae such as Anisakidae, Opisthorchis, and other helminths. In Kazakhstan, specific freezing regimes have been established and must be strictly followed at production facilities: at −40 °C, a minimum of 7 h is required to ensure complete inactivation of larvae within the fish tissue; at −35 °C, 14 h are necessary; and at −28 °C, 32 h are required [63]. It is essential that these temperatures are reached throughout the entire mass of the fish, as parasites may survive in inadequately frozen areas. At transportation and processing facilities (e.g., freezer vessels), temperature control down to −18 °C or lower, along with compliance with sanitary storage standards, is mandatory [64].

Dead parasites do not pose an infectious risk to consumers; however, their presence remains a significant problem for product quality. They cause visible defects such as nodules, dark spots, and granulomas, which negatively affect the organoleptic properties of fish and result in economic losses due to culling or reduced market value of filets. In some cases, even non-invasive forms of parasites are associated with postmortem tissue changes, including degradation, which further compromises product quality [65,66]. Thus, the presence of dead parasites lowers consumer appeal and presents a challenge for the food industry, despite the absence of infectious risk.

This study presents the first comprehensive molecular screening of helminths in imported fish products on the Kazakhstani market, encompassing 19 fish species from diverse geographical origins. Our findings indicate a high prevalence of zoonotic nematodes, particularly *A. simplex* and *A. pegreffii*, in alignment with earlier reports from other regions. The presence of these parasites confirms the ongoing risk of anisakiasis associated with the consumption of undercooked or raw fish.

Infection intensity varied significantly among fish species. *H. stelleri* (greenling) exhibited the highest mean intensity, with an average of 82.9 helminths per fish (SD = 58.1), ranging from 24 to 232 individuals. *S. scombrus* (mackerel) and *M. poutassou* (blue whiting) followed, with mean intensities of 46.1 (SD = 41.2) and 22.7 (SD = 27.4), respectively. Notably, a single specimen of *G. morhua* (Atlantic cod) harbored 240 *E. gadi* helminths, highlighting the potential for localized heavy infections even in species with low overall prevalence.

According to Levsen et al. (2022), anisakid nematodes such as *Anisakis simplex* and *Pseudoterranova decipiens* were predominant in gadid fishes from the southern Barents Sea, which aligns with our findings in cod, haddock, and blue whiting imported from this region [5]. In our study, *H. stelleri* (greenling) demonstrated the highest prevalence of infection (97.1%). This observation is consistent with previous reports from the Pacific Ocean, where high infection rates with larval stages of *Anisakis* spp. and *Hysterothylacium* spp. have been frequently documented [67]. Notably, the prevalence of helminth infection in *M. poutassou* (blue whiting) reached 88.1%, significantly exceeding values reported in other Atlantic studies, where prevalence rarely surpasses 50% [68]. This discrepancy may be attributable to differences in sanitary handling, post-harvest processing, or transport and storage conditions during importation to Kazakhstan. *O. eperlanus* (smelt), a species native to northern waters, exhibited a relatively low infection rate of 6.8%. This finding is in line with previous data from Sakhalin, where osmerid fishes were found to exhibit selective parasitism with specific helminth species [19]. The identification of cestodes (*E. crassum*) and acanthocephalans (*E. gadi*) further illustrates the diversity of helminths present in imported fish. These parasites have also been commonly reported in marine fish from Russian and Icelandic waters [7].

The molecular methods employed in this study, PCR using species-specific primers followed by sequencing, proved to be highly sensitive and allowed for precise species-level identification, including the detection of mixed infections. This approach markedly surpasses traditional morphological diagnostics in accuracy and resolution. Similar conclusions were drawn by Suthar (2021), who highlighted the limited application of molecular tools in regions with underdeveloped epidemiological frameworks [69].

The *N. surmenicola* isolates obtained in this study (PV776615.1, PV776612.1, PV776614.1) correspond to individual taxa that form a well-supported clade, combined with other genetically identical sequences of the same species from the GenBank database (Figure 5). This confirms the identity of the obtained samples and their belonging to *N. surmenicola*, which is also consistent with previously published data (KU512308.1, JN662466.1, FJ572929.1). The closest related species within the genus are *N. sphynae* and *N. africana*, which form a sister group with them. The *H. trichiuri* isolates (PV776619.1 and PV776636.1) obtained within the framework of this study formed a separate clade with previously registered sequences from the database (MT823199.1, MT823200.1, FJ572943.1), which confirms that these samples belong to the species *H. trichiuri*. Currently, only nine sequences of this species (including those from this study) have been registered in the GenBank database, which were found in the keeltail pomfret and the squid [70]. The isolates of the genus *Eubothrium* obtained in this study (PV776617.1 and PV776620.1) correspond to two genetically distinct lineages. The first isolate (05-Sa-Ec-1 and 05-Sa-Ec-3) is grouped with *E. crassum* (AF286947.1, KR780880.1, PQ570012.1) with high support (bootstrap = 98%), confirming their identity. Interestingly, *E. fragile* (KR780991.1) and *E. rugosum* (KR780914.1) occupy a separate, more basal position, which confirms the phylogenetic isolation of this species.

The largest cluster comprises isolates of *A. simplex, A. pegreffii,* and *A. ziphidarum*, which grouped into several moderately supported subclades (bootstrap support ranging from 36% to 44%) (Figure 6). Isolates from the present study were interspersed among GenBank reference sequences, confirming their identities as *A. simplex* or *A. pegreffii*. Notably, *A. ziphidarum* isolates (PV770758.1 and PV770860.1) formed a separate, weakly supported branch (25%), indicating potential divergence from other *Anisakis* spp. For the *Pseudoterranova* species, a distinct subclade comprises *P. decipiens, P. cattani*, and *P. azarasi*, with weak to moderate bootstrap support (28–78%). Several isolates from this study (e.g., PV771506.1, PV771507.1, PV775360.1) grouped within the *P. decipiens* clade, validating their species assignment. The clustering suggests a relatively close relationship among members of the *Pseudoterranova* complex, albeit with limited genetic resolution based on *ITS* alone. The isolate PV554186.1 from this study grouped with *H. aduncum* reference sequence KF923932.1 (bootstrap 35%), confirming its taxonomic placement within this genus. The clade *C. osculatum* includes isolates with moderate bootstrap support (31–37%). Study-derived isolates (PV771551.1, PV771524.1, PV771522.1, PV770256.1) clustered closely with reference sequences (KY659384.1, MT258502.1), supporting their identification. The relatively low internal support may reflect intraspecific variation or insufficient phylogenetic signal from the *ITS* region.

The *E. gadi* clade formed a well-defined group within the genus *Echinorhynchus*, sharing a close evolutionary relationship with *E. bothniensis* and *E. hexagrammi* (Figure 7). These results are consistent with earlier findings suggesting that these species are genetically proximate and may share overlapping ecological niches, particularly in marine and anadromous fish hosts [71,72,73]. Other species within the genus, such as *E. truttae, E. brayi*, and *E. cinctulus*, were more distantly related but remained within the broader *Echinorhynchus* lineage. The placement of *Polymorphus* species as an outgroup further supports the monophyly of *Echinorhynchus* and underscores the genetic divergence between these acanthocephalan genera.

Regarding human infections with the identified parasite species, such data for Kazakhstan are not available in open-access sources, and the diagnostic protocols in the country’s medical laboratories do not typically include screening for *Anisakis* spp. or related parasites. As for clinical cases in geographical regions where the imported fish were caught, available data suggest a limited yet plausible correlation between the consumption of raw or undercooked fish, “high-risk” carrier species, and the occurrence of anisakiasis. However, interpretation is complicated by substantial underreporting and variability in case registration systems across countries.

For instance, based on insurance registry data, the average annual incidence of anisakiasis in Japan during 2018–2019 was estimated at 19,737 cases, with *Anisakis simplex sensu stricto* molecularly confirmed in 88.4% of patients [74]. In Europe, the highest disease burden has been documented in Spain, where a 19-year analysis of hospitalizations demonstrated sustained disease circulation and clinical relevance, including allergic manifestations [75]. At the “product type” level, recurring associations have been observed: in Western Europe, clinical cases are frequently linked to herring (*C. harengus*), whereas in the Mediterranean region, pickled anchovy (*Engraulis encrasicolus*) and other traditional raw fish products are more commonly implicated. In Italy, both gastro-allergic and intestinal forms of anisakiasis have been reported following the consumption of pickled anchovies [76,77]. Numerous clinical case series and reviews, some involving endoscopic extraction of larvae, have also been published in South Korea, reflecting a high prevalence linked to raw food traditions. Allergic reactions are not uncommon in these cases as well [78,79].

It should be noted that our study revealed the highest prevalence of *Anisakis* spp. infections, detected in 74% of the examined imported fish species. This finding represents a direct threat to public health in Kazakhstan. If proper shock-freezing protocols are not strictly followed, the ingestion of live anisakid larvae can lead to serious clinical outcomes, including intestinal obstruction [80], immunomodulatory and potentially carcinogenic effects on intestinal organoids [81], and even early-stage gastric cancer [82]. Moreover, there is growing evidence of a direct link between allergic reactions and the consumption of well-cooked fish infected with *Anisakis* larvae [83]. *Anisakis* spp. produces more than 14 allergenic compounds, several of which are heat-stable. As a result, allergic responses have been reported even after eating thermally processed fish [76,84].

Differences in infection rates among fish species are expected and can be attributed to a combination of ecological and methodological factors. Trophic ecology and position in the food chain play a key role: species that feed at higher trophic levels or consume infected prey tend to accumulate more parasite larvae. Parasite aggregation typically increases with the number of predators and prey involved in transmission, leading to higher prevalence and intensity in certain hosts compared to others [85]. Additionally, fish age and size are strongly correlated with parasite infection rates. As fish grow older and larger, their cumulative exposure to infective stages increases, and they tend to consume larger prey items. Body length has been identified as one of the strongest predictors of larval parasite abundance in several species, including cod and horse mackerel [5]. Other important factors include changes in diet and habitat use, whether pelagic, demersal, or deep-sea environments, which influence the frequency of encounters with infected intermediate hosts, such as crustaceans or small fish, and contribute to interspecific variation in infection levels [86]. Finally, spatial and temporal factors such as geography, fishing area, and season also significantly influence infection patterns. The same fish species may exhibit different infection levels depending on the region or season, reflecting spatial variability in parasite circulation and host community composition, including the presence and activity of definitive hosts such as marine mammals [76,87].

Seafood markets in Central Asia are characterized by a high dependence on imports, with a significant portion of fish products originating from international suppliers. According to World Integrated Trade Solution data, the main exporters of fish to Uzbekistan include Lithuania, Norway, Vietnam, China and Indonesia [88]. Kyrgyzstan primarily imports fish from Russia, China, Norway, Kazakhstan and Iceland [89], while Tajikistan receives fish products mainly from Russia, Vietnam, and Kazakhstan, with additional imports from Belarus and Uzbekistan [90,91]. In contrast, Turkmenistan relies largely on domestic fish production and aquaculture in the Caspian Sea basin, and available data on its imports are limited. Given Norway’s position as a major exporter of fish to the region, the detection of zoonotic parasites in Norwegian fish products, as demonstrated in our study, raises important concerns for regional food safety. These findings highlight the potential risks within transboundary supply chains and emphasize the need for regular parasitological monitoring using molecular diagnostic tools. Implementing such surveillance measures can support timely detection of pathogens, reduce public health risks, and strengthen food safety regulation across Central Asian markets.

## 5. Conclusions

This study presents the first comprehensive molecular survey of helminths in imported fish products on the Kazakhstani market, revealing a high prevalence of zoonotic parasites, particularly *Anisakis* spp., in several commercially important species. The use of molecular diagnostic tools, such as PCR and DNA sequencing, significantly improved the accuracy of parasite detection, confirmed mixed infections, and enhanced phylogenetic resolution. These findings highlight the parasitological burden in imported marine fish and underscore the public health risks associated with consuming raw or undercooked seafood. In light of increasing fish imports and the growing popularity of minimally processed products, regular monitoring using advanced molecular techniques should be integrated into national food safety programs to safeguard consumer health and support sustainable seafood trade.

## Figures and Tables

**Table 1:** Morphometric parameters and origin of the studied commercial fish species.

№	Fish Species	Length, cm	Weight, g	Importing Countries	Origin
1	mackerel (*S. scombrus*)	31.7 ± 7	349.2 ± 50	Norway, Iceland, UK, EU Atlantic coasts.	Wild capture.
2	capelin (*M. villosus*)	17.1 ± 3	34.2 ± 12	Iceland, Norway, Barents Sea (Russia).	Wild capture.
3	herring (*C. harengus*)	36.9 ± 4	376.7 ± 70	Norway, Iceland, North Sea, Baltic.	Wild capture.
4	pollock (*T. chalcogramma*)	37.9 ± 9	352.0 ± 100	Russia (Far East).	Wild capture.
5	flounder (*P. flesus*)	32.6 ± 4	833.4 ± 120	Baltic Sea, North Sea (Norway, Denmark, Poland).	Wild capture.
6	char (*S. alpinus*)	46.5 ± 6	1255.3 ± 200	Iceland, Norway.	Both wild capture and aquaculture.
7	cod (*G. morhua*)	60.5 ± 10	3088.86 ± 250	Norway, Iceland, Russia, Canada.	Wild capture.
8	blue whiting (*M. poutassou*)	27.8 ± 5	162.4 ± 40	Norway, Iceland, EU Atlantic.	Wild capture.
9	smelt (*O. eperlanus*)	15.7 ± 3	39.8 ± 15	Baltic Sea (Estonia, Latvia, Russia).	Wild capture.
10	haddock (*M. aeglefinus*)	58.6 ± 4	1583.6 ± 200	Norway, Iceland, Barents Sea, North Sea.	Wild capture.
11	amberjack (*S. quinqueradiata*)	72.4 ± 10	5358.6 ± 500	Japan.	Aquaculture.
12	greenling (*H. stelleri*)	44.5 ± 9	1327.1 ± 200	Russia (Far East, Sea of Japan), Japan.	Wild capture.
13	sprat (*C. cultriventris*)	11.3 ± 2	12.5 ± 7	Caspian Sea (Russia, Azerbaijan).	Wild capture.
14	sea bass (*D. labrax*)	40.2 ± 7	862.7 ± 150	Greece and Turkey	Aquaculture.
15	dorado (*S. aurata*)	31.5 ± 8	799.7 ± 200	Greece, Turkey, Egypt.	Aquaculture.
16	sole (*S. solea*)	34.6 ± 10	745.1 ± 179	North Sea (Netherlands, UK).	Wild capture.
17	grouper (*S. babcocki*)	37.67 ± 8	954 ± 200	Turkey, China.	Both wild capture and aquaculture
18	pink salmon (*O. gorbuscha*)	47.8 ± 5	1421.6 ± 250	Russia (Far East)	Wild capture.
19	salmon * (*S. salar*)	-	-	Norway, Chile.	Aquaculture

* In the market, salmon is offered solely as filets.

**Table 2:** Primer design and amplification parameters.

Primer Name	Type of Helminths	Target Gene	Sequence	Parameters	References
NC13/NC2	Nematoda	5.8S	F: 5′-ATCGATGAAGAACGCAGC-3′ R: 5′-TTAGTTTCTTTTCCTCCGCT-3′	95 °C 4 min, (94 °C 30 s, 60 °C 30 s, 72 °C 45 s) 40×, 72 °C 7 min	[44]
NC5/NC2	Nematoda	ITS-2	F:5′-GTAGGTGAACCTGCGGAAGGATCATT-3′ R:5′-TTAGTTTCTTTTCCTCCGCT-3′	95 °C 4 min, (94 °C 30 s, 60 °C 30 s, 72 °C 45 s) 40×, 72 °C 7 min	[45]
SSU_F_04/SSU_22_R	Nematoda	18S	F: 3′-GCTTGTCTCAAAGATTAAGCC-5′ R:5′-ATGTGGAGCCGTTTATCAGG-3′	95 °C 2 min, (95 °C 1 min, 57 °C 45 s, 72 °C 1 min) 30×, 72 °C 10 min	[46]
AcanCoI_F/ AcanCoI_R	Acanthocephala	COI	F: 3′-TTCTACAAATCATAARGATATYGG-5′ R:5′-AAAATATAMACTTCAGGATGACCAAA-3′	94 °C 7 min, (94 °C 30 s, 48 °C 1 min and 75 °C 1 min) 40×, 75 °C 10 min	[47]
JB3/JB4,5	Cestoda	CO1	F:5′-TTTTTTGGGCATCCTGAGGTTTAT-3′ R:5′-TAAAGAAAGAACATAATGAAAATG-3′	95 °C 5 min, (95 °C 50 s, 50 °C 50 s, 72 °C 50 s) 35×, 72 °C 5 min	[48]
Nyb-28S-F/ Nyb-28S-R	Cestoda	28S	F: 5′-TAGGTCGACCCGCTGAACTTA-3′ R: 5′-GCATAGTTCACCATCTTTCGG-3′	94 °C 2 min, (94 °C 30 s, 55 °C 45 s, 72 °C 2 min) 32×, 72 °C 10 min	[49]

**Table 3:** Prevalence and Intensity of Helminths Identified in Imported Fish.

№	Species of the Studied Fish	N Examined/ N Infected	% Prevalence (95% CI)	N Helminths Found	Range of Intensity	Mean (SD) Intensity	Helminth Species Identified
1	mackerel (*S. scombrus*)	50/46	92.0 (80.7–97.8)	969	1–190	46.1 (41.2)	*A. simplex*, *A. pegreffii*
2	capelin (*M. villosus*)	71/27	38.0 (26.7–50.3)	44	1–4	1.6 (0.8)	*Anisakis*, *Contracaecum osculatum*, *A. pegreffii*
3	herring (*C. harengus*)	50/48	96.0 (86.3–99.5)	565	1–34	11.8 (8.2)	*A. simplex*, *A. pegreffii*
3	pollock (*T. chalcogramma*)	50/31	62.0 (47.1–75.3)	123	1–50	4.0 (8.3)	*A. simplex*, *A. pegreffii*, *C. osculatum*
		50/17	34.0 (21.1–48.8)	111	1–15	6.5 (4.4)	*Echinorhynchus gadi*
4	hake (*M. merluccius*)	50/29	58.0 (43.2–71.8)	191	1–28	6.6 (6.9)	*Hepatoxylon trichiuri*
		50/3	6.0 (1.2–16.6)	5	1–3	1.7 (1.2)	*A. simplex*, *A. pegreffii*, *A. ziphidarum*
5	flounder (*P. flesus*)	50/8	16.0 (7.2–29.1)	7	1–3	0.9 (0.7)	*Anisakis* spp.
6	char (*S. alpinus*)	50/21	42.0 (28.2–56.8)	74	1–14	3.5 (3.5)	*Eubothrium crassum*
		50/11	22.0 (11.5–36.0)	47	1–15	4.3 (4.7)	*A. simplex*, *A. pegreffii*, *Hysterothylacium aduncum*
7	cod (*G. morhua*)	10/2	20.0 (2.5–55.6)	7	1–5	3.5 (2.1)	*Nybelinia surmenicola*
		10/4	40.0 (12.1–73.8)	50	1–19	12.5 (7.4)	*A. simplex*, *A. pegreffii*
		10/1	10.0 (0.2–44.5)	240	240	-	*Echinorhynchus gadi*
8	blue whiting (*M. poutassou*)	59/52	88.1 (77.1–95.1)	1178	3–151	22.7 (27.4)	*A. simplex*, *A. pegreffii*, *H. aduncum*
9	smelt (*O. eperlanus*)	44/3	6.8 (1.4–18.7)	3	1–1	1.0 (0.0)	*Pseudoterranova decipiens*
10	haddock (*M. aeglefinus*)	20/3	15.0 (3.2–37.9)	6	1–3	2.0 (1.0)	*H. aduncum*, *A. simplex*
11	amberjack (*S. quinqueradiata*)	32/4	12.5 (3.5–29.0)	7	1–2	1.8 (0.5)	*A. simplex*
12	greenling (*H. stelleri*)	35/34	97.1 (85.1–99.9)	2819	24–232	82.9 (58.1)	*A. simplex*, *A. pegreffii*
		35/12	34.3 (19.1–52.2)	320	4–86	26.7 (23.3)	*Nybelinia surmenicola*
13	sprat (*C. cultriventris*)	76/-	-	-	-	-	-
14	sea bass (*D. labrax*)	20/-	-	-	-	-	-
15	dorado (*S. aurata*)	22/-	-	-	-	-	-
16	sole (*S. solea*)	10/-	-	-	-	-	-
17	grouper (*S. babcocki*)	15/5	33.3 (11.8–61.6)	38	3–16	7.6 (5.3)	*Anisakis simplex*
18	pink salmon (*O. gorbuscha*)	4/3	75.0 (19.4–99.4)	32	2–26	10.7 (11.4)	*Anisakis simplex*
19	salmon (*S. salar*)	7/-	-	-	-	-	-

95% CI: 95% confidence interval; SD: standard deviation; N: number.

## Data Availability

The original contributions presented in this study are included in the article. Further inquiries can be directed to the corresponding author.

## Abbreviations

The following abbreviations are used in this manuscript:

N	number
bp	base pairs
CI	confidence interval
cox1	cytochrome c oxidase subunit 1
DNA	deoxyribonucleic acid
F	Forward
ML	Maximum Likelihood
nad1	NADH dehydrogenase subunit 1
OR	Odds Ratio
R	Revers
SD	standard deviation
TBE	Tris-borate-EDTA buffer
USA	United States of America
UV	ultraviolet
